# Neonatal erythropoiesis and subsequent anemia in HIV-positive and HIV-negative Zimbabwean babies during the first year of life: a longitudinal study

**DOI:** 10.1186/1471-2334-6-1

**Published:** 2006-01-03

**Authors:** Melissa F Miller, Jean H Humphrey, Peter J Iliff, Lucie C Malaba, Nkosinathi V Mbuya, the ZVITAMBO Study Group, Rebecca J Stoltzfus

**Affiliations:** 1 Cancer Prevention Fellowship Program, Division of Cancer Prevention, National Cancer Institute, Bethesda, MD, USA; 2 Center for Human Nutrition, Department of International Health, Johns Hopkins Bloomberg School of Public Health, Baltimore, MD, USA; 3 Paediatrics and Child Health, University of Zimbabwe, Harare, Zimbabwe; 4 Division of Nutrition, Institute of Food, Nutrition and Family Sciences, University of Zimbabwe, Harare, Zimbabwe; 5 Division of Nutritional Sciences, Cornell University, Ithaca, NY, USA; 6 Members of the ZVITAMBO Study Group, in addition to the named authors are: Henry Chidawanyika, Agnes Mahomva, Florence Majo, Edmore Marinda, Michael Mbizvo, Lawrence Moulton, Kuda Mutasa, Mary Ndhlovu, Robert Ntozini, Ellen Piwoz, Lidia Propper, Philipa Rambanepasi, Andrea Ruff, Naume Tavengwa, Brian Ward, Lynn Zijenah, Claire Zunguza, Partson Zvandasara; principal investigators are Kusum Nathoo and Jean Humphrey

## Abstract

**Background:**

Anemia is common in HIV infection and independently associated with disease progression and mortality. The pathophysiology of HIV-related anemia is not well understood especially in infancy.

**Methods:**

We conducted a longitudinal cohort study nested within the Zimbabwe Vitamin A for Mothers and Babies Project. We measured hemoglobin, erythropoietin (EPO), serum transferrin receptor (TfR) and serum ferritin at 6 weeks, 3 and 6 months of age and hemoglobin at 9 and 12 months in 3 groups of randomly selected infants: 136 born to HIV-negative mothers, and 99 born to HIV-positive mothers and who were infected themselves by 6 weeks of age, and 324 born to HIV-positive mothers but who did not become infected in the 6 months following birth.

**Results:**

At one year of age, HIV-positive infants were 5.26 (adjusted odds ratio, P < 0.001) times more likely to be anemic compared to HIV-negative infants. Among, HIV-negative infants, EPO was or tended to be inversely associated with hemoglobin and was significantly positively associated with TfR throughout the first 6 months of life; TfR was significantly inversely associated with ferritin at 6 months; and EPO explained more of the variability in TfR than did ferritin. Among infected infants, the inverse association of EPO to hemoglobin was attenuated during early infancy, but significant at 6 months. Similar to HIV-negative infants, EPO was significantly positively associated with TfR throughout the first 6 months of life. However, the inverse association between TfR and ferritin observed among HIV-negative infants at 6 months was not observed among infected infants. Between birth and 6 months, mean serum ferritin concentration declined sharply (by ~90%) in all three groups of babies, but was significantly higher among HIV-positive compared to HIV-negative babies at all time points.

**Conclusion:**

HIV strongly increases anemia risk and confounds interpretation of hematologic indicators in infants. Among HIV-infected infants, the EPO response to anemia is attenuated near the time of infection in the first weeks of life, but normalizes by 6 months.

## Background

By the end of 2003, an estimated 2.1 million children worldwide were living with HIV [1]. Almost all infected children acquire HIV from their mother *in utero*, during delivery or during breastfeeding. Nearly 90% of pediatric infections occur in sub-Saharan Africa, where many countries report that more than one in five pregnant women visiting antenatal clinics are HIV-infected. In the absence of antiretroviral therapy, nearly one-third of vertically infected children living in the developed world rapidly progress to AIDS or death in the first year of life [2]. It is likely that HIV disease progression is rapid in resource-poor countries because of the high prevalence of other childhood infections and nutrient deficiencies. Anemia is a common complication of HIV infection and is independently associated with disease progression and mortality [3,4]. The pathophysiology of HIV-related anemia is not well understood and may be especially complicated amidst the dynamic changes associated with normal hematological development in early infancy.

Anemia in HIV-infected adults has the characteristics of the "anemia of chronic disease" [5] and is associated with an impaired erythropoietin (EPO) response to anemia [6] and failure to mobilize iron from liver stores [7]. It is believed that proinflammatory cytokines may play a role in the suppression of erythropoiesis during HIV infection [8-10]. However, in a recent study among 12-month old HIV-infected Malawian infants, EPO response to anemia was not different than the response in uninfected infants [11]. Other factors that may contribute to anemia during HIV infection include the direct effect of HIV-1 on bone marrow cells, adverse reactions to antiretroviral drugs, opportunistic infections and neoplasms infiltrating the bone marrow, vitamin B12 or iron deficiency, autoimmune hemolytic anemia, circulating anti-EPO autoantibodies, and other coexisting medical conditions [5].

**Figure 1:**
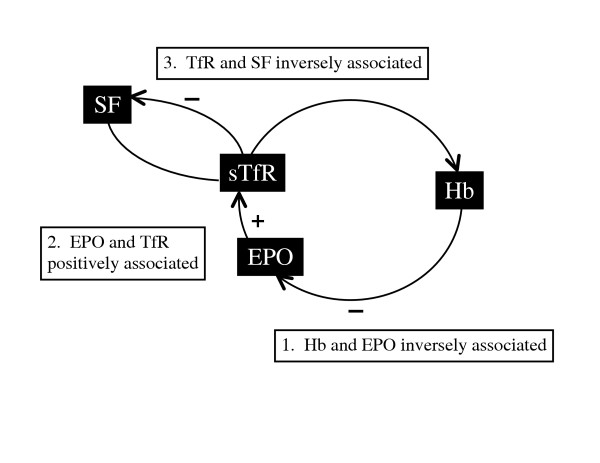
**Model of the relationships between indicators or iron status and erythropoiesis**. The model is based on the assumptions that hypoxia determines EPO production which in turn stimulates erythropoietic activity that drives the need for iron and corrects the hypoxia. Abbreviations: Hb, hemoglobin; EPO, erythropoietin; TfR, transferrin receptor; SF, serum ferritin.

For the present study, we used a model of the relationships between indicators of iron status and erythropoiesis (Figure 1) to guide our examination of erythropoiesis in young infants and the impact of HIV infection on the process. EPO is secreted by the kidney in response to hypoxia [12] inducing the bone marrow to synthesize more red blood cells and maintaining their viability as they differentiate [13,14]. Provided there is a normal EPO-generating apparatus in the kidney, plasma EPO will increase exponentially as circulating hemoglobin decreases, which in the presence of a sufficient iron reserve, increases the number of red cells and corrects the hypoxia. Transferrin receptors (TfRs) present on the surface of erythroid cells increase with the expansion of the erythroid mass, and a soluble truncated form of the tissue receptor increases proportionally in the serum [15-17].

During iron deficiency, ferritin levels decrease. The number of TfRs per cell increases in an effort to gather more iron, and circulating levels of TfR increase. According to this model, TfR levels do not distinguish between functional iron deficiency (more TfRs per cell) and an increase in red cell number (simply more cells, i.e., erythropoiesis). We hypothesized that, in HIV-uninfected babies, the following relationships would be found: (1) EPO would be inversely associated with hemoglobin, reflecting the erythropoietic response to anemia; (2) EPO would be positively associated with TfR, reflecting the erythroid expansion in response to EPO; (3) ferritin would be inversely associated with TfR, reflecting the depletion of iron stores when erythropoiesis is stimulated in the presence or absence of adequate iron stores; and (4) at 6 weeks and 3 months, the EPO concentration would explain more variability in TfR concentration than ferritin (i.e., the association between TfR and EPO would be stronger than the association between TfR and ferritin). At 6 months, the approximate age at which iron can become a limiting nutrient in normal birthweight infants, ferritin concentration would explain more variability in TfR than EPO, and ferritin would be positively associated with hemoglobin, reflecting the relation between iron stores and hemoglobin production.

We expected that HIV infection would modify these relationships, but there was insufficient evidence about the specific perturbations in the regulation of erythropoiesis to posit hypotheses. Among HIV-negative infants born to HIV-positive mothers, we speculated that the indicators of erythropoiesis may be transiently perturbed during early infancy, but would normalize with infant age.

We conducted a longitudinal cohort study nested within the Zimbabwe Vitamin A for Mothers and Babies Project (ZVITAMBO). The specific aims of the nested study were: (1) to describe the course of changes in hemoglobin and other indicators of anemia and iron status in a group of breast-fed Zimbabwean infants, stratified by maternal and infant HIV status; (2) to examine the validity of this model (see Figure 1) during early infancy by testing our hypotheses at 6 weeks, 3 and 6 months of age among HIV-positive babies and HIV-negative babies of HIV-positive and negative mothers. From these analyses, we make inferences about the usefulness of TfR as an indicator of iron deficiency in this age group and the pathophysiology of anemia in these babies according to their HIV status.

## Methods

### Study population

Subjects were drawn from participants of ZVITAMBO, which enrolled 14,110 mother-baby pairs within 96 hours of delivery from maternity clinics and hospitals in Harare between November 1997 and January 2000 to study the impact of maternal and neonatal vitamin A supplementation on several outcomes [18-20]. Pairs were eligible if neither the mother nor the baby had an acutely life threatening condition and the infant was a singleton with birthweight >1500 g. Written informed consent was obtained from the mother. At the time the study was carried out, neither vitamin A supplementation nor HIV antiretroviral therapy was part of routine perinatal or postnatal care. Among infants enrolled beginning in October 1998, a 34% sub-sample (seven of every eight born to HIV-positive mothers and one of every ten born to HIV-negative mothers) was randomly selected for the anemia substudy, to yield an adequate sample size among HIV-positive and HIV-negative infants to detect a 5 g/L difference in hemoglobin concentration. Babies who had received a blood transfusion during the neonatal period were excluded from the anemia substudy. Only babies assigned to the placebo control group (i.e., both mother and baby received placebo) were included in the present analysis for a total of 136 and 575 babies born to HIV-negative and HIV-positive mothers, respectively. The ZVITAMBO trial protocol was approved by the Medical Research Council of Zimbabwe (MRC-Z), the Committee on Human Research of The Johns Hopkins Bloomberg School of Public Health (CHR-JHBSPH), the Medicines Control Authority of Zimbabwe, and the Montreal General Hospital Research Ethics Committee. The MRC-Z and the CHR-JHBSPH provided further approval to conduct additional laboratory analyses for this anemia substudy.

### Procedures

At recruitment, demographic and obstetric information was collected by maternal history or transcription from the medical record. Gestational age was estimated using the method of Capurro [21]. Birthweight was measured within 96 hours of delivery to the nearest 5 g using an electronic scale (Seca model 727, Hanover, MD). Mothers and babies were followed at 6 weeks, 3 months and thereafter 3 monthly up to 24 months of age in a study clinic or at home. Detailed infant feeding information, including breast-feeding status and whether or not any of 22 nonmilk liquids, nonhuman milks (animal milks and commercial formula), medicines (traditional fluids, oral rehydration salts, other prescribed), or solid foods had ever been given to the infant were collected at enrolment, 6 weeks, 3 months, and 6 months after delivery.

Medical care, including treatment of acute illness, and counseling for pre- and post- HIV test, psycho-social support, and infant feeding, were offered throughout the trial; travel costs to the follow-up clinic were reimbursed.

### Blood collection and processing

At baseline and each follow-up visit, study midwives collected maternal and infant blood by venipuncture or heelstick (capillary) into clot and EDTA tubes. Maternal and infant serum and plasma were prepared and stored at -70°C within two hours of phlebotomy. Aliquots of infant whole blood were refrigerated and cell pellets prepared (Amplicor whole blood PCR sample preparation method; Roche Diagnostics Systems, Alameda, CA, USA) generally within a few days of collection and stored at -70°C. Aliquots of baseline maternal serum were refrigerated.

### Maternal HIV testing

Refrigerated maternal serum was tested for HIV status by two ELISA assays for detecting antibody to both HIV 1 and 2 [GeneScreen (Sanofi Diagnostics Pasteur PRx, Johannesburg, South Africa) and Murex (Murex Diagnostics, Johannesburg, South Africa)] run in parallel. Discordant ELISA results were resolved by Western Blot. CD4 cells from maternal baseline samples that tested HIV positive were enumerated using a Facscount (Becton Dickinson International, Erembodegem, Belgium) within 48 hours of phlebotomy.

### Infant HIV diagnosis

After all patient contact was completed, the last available infant specimen was tested for HIV (plasma by ELISA for samples collected at >18 months; pellets by Amplicor HIV-1 DNA test version 1.5 (Roche Diagnostic Systems) for samples collected at younger ages). If this sample was HIV-negative, the baby was classified as negative and no further testing was carried out. If the last sample was HIV-positive, samples collected at intervening time points were tested to determine the timing of infection.

### Hemoglobin and iron parameter assays

Hemoglobin was measured on whole blood samples on the day of collection for all mothers at baseline and for all infants in the anemia substudy at baseline and visits up to 12 months using the HemoCue hemoglobinometer (HemoCue, Mission Viejo, CA). Daily quality control of the instrument was performed. Hemoglobin data were unavailable if the baby missed the study visit, or if the blood sample was insufficient as a result of the mother's refusal for her baby to give blood, inadequate sample volume, sample clotting, or failure by the laboratory to perform the test before the end of the day in which the specimen had been drawn.

We measured TfR, ferritin and EPO concentrations in plasma (or serum when plasma was unavailable). TfR concentrations were measured by enzyme immunoassay (Ramco Laboratories, Houston, TX) (intra-assay coefficient of variation, 3.9% at 6.7 mg/L and 2.7% at 13.2 mg/L; inter-assay coefficient of variation, 8.8% and 6.6%, respectively); ferritin by enzyme-linked immunoassay (Ramco Laboratories) (intraassay coefficient of variation, 4.0% at 70 μg/L and 4.7% at 300 μg/L; inter-assay coefficient of variation, 9.6% and 9.2%, respectively); and EPO using the Quantikine In Vitro Diagnostic EPO ELISA (R&D Systems, Minneapolis, MN) (intra-assay coefficient of variation, 2.3% at 42 IU/L and 2.4 at 13 IU/L; inter-assay coefficient of variation, 5.2% and 3.4%, respectively).

### Definitions

The criteria we used to diagnose anemia were modified from those of the World Health Organization [22]. Several studies have suggested that during infancy, the WHO cut-off value of 110 g/L may overestimate anemia [23-25]; therefore, we used a more stringent cut-off value of 105 g/L [26]. We did not define anemia in infants younger than 3 mo of age, because hemoglobin does not reflect iron status in early infancy but reflects a normal physiologic decline in concentration. Low plasma ferritin concentration is considered to be an accurate indicator of iron stores in the liver [27,28]. We used the WHO standard of 12 μg/L to indicate absent or depleted iron stores in infants aged up to 6 months.

### Statistical analysis

Plasma EPO, TfR, and ferritin concentrations were normalized by log transformation; geometric means (95% CI) are reported. Differences across HIV status groups in baseline characteristics and indicators of anemia and iron status were examined by ANOVA for continuous variables and the chi-squared test of homogeneity for categorical variables. We used multivariate linear and logistic regression analysis to compare hemoglobin values and the proportion of anemic children across HIV status groups and adjusted for birthweight in these models. The association between indicators of anemia and iron status was examined by linear regression and partial correlation coefficients. The relevant interaction terms between the independent variable and HIV status groups were included in regression models to examine differences in the association between indicators by HIV status group. Adjusting for birthweight in these models did not change the conclusions, and, therefore, was not included. We performed a likelihood ratio test to determine the overall statistical significance of the interaction between HIV status and the independent variable. For all analyses, we set the significance levels at α = 0.05. A Bonferroni correction was used to determine which associations were unlikely to be the result of chance alone.

We created three categories of babies: the 136 babies born to HIV-negative mothers (Nn group) and two groups of babies born to HIV-positive mothers: (1) babies who tested PCR negative at 6 months or later (Pn group, n = 324); and (2) babies who tested PCR-positive at baseline or 6 weeks (Pp group, n = 99). Babies who did not fall neatly into these three groups were excluded from the analysis. These included those who tested PCR-positive for the first time at 3 or 6 months (n = 37), and babies with an indeterminate HIV status (n = 115). Babies were classified as HIV-indeterminate if maternal baseline HIV status was unknown (n = 2) or maternal baseline status was positive and no results were available for the baby (n = 2), the baby's last available PCR result was prior to 6 months of age and was negative (n = 108), or the baby's PCR results were inconsistent such that a negative result followed a positive result (n = 3). Only 4 infants in the Nn group and 3 in the Pn group died by 12 months of age, but among Pp infants, 33 (33.3%) had died by 6 months of age and 51 (51.5%) by 12 months.

We carried out the analysis only on the available data, and did not impute values for missing observations. Hemoglobin values >160 g/L at 6 weeks (n = 1), 3 months (n = 1) and 6 months (n = 4) were excluded from the analysis. All analyses were performed with the use of Stata (verson 8.0, Stata Corporation, College Station, TX).

## Results

Compared to HIV-negative mothers (Nn), HIV-positive mothers (Pn and Pp) had lower hemoglobin concentrations and were more likely to be anemic, older and of higher parity (Table 1). Among HIV-positive mothers, those whose babies remained PCR-negative at 6 months (Pn) had higher CD4 counts than those whose babies were PCR-positive by 6 weeks (Pp). Babies who were PCR-positive for HIV by 6 weeks (Pp) had lower mean birthweight and mean length.

**Table 1:** Characteristics of Zimbabwean newborns by HIV status of the mother and her infant.

	HIV status group			
	Nn (*n *= 136)	Pn (*n *= 324)	Pp (*n *= 99)	P-value*

*Maternal characteristics*				
Hemoglobin, g/L^†, ‡^	122 (17)^a^	113 (18)^b^	110 (19)^b^	<0.001
>110, %	78.8	60.6	53.2	
90–110, %	18.9	29.8	35.1	
<90, %	2.2	9.6	11.7	<0.001
CD4+ lymphocyte count, cells/μL				
≥400	-------	40.7	34.3	
200–400		33.6	29.2	
<200		12.3	26.2	<0.01
Age, y	23 (5)^a^	26 (5)^b^	25 (5)^b^	<0.0001
Parity	1.9 (1.2)^a^	2.4 (1.3)^b^	2.3 (1.3)^b^	<0.001
*Infant characteristics*				
Sex, % female	48.5	47.5	50.5	0.87
Birthweight, g	2917 (454)^a^	2969 (464)^a^	2830 (462)^b^	0.030
<2500 g, %	19.2	14.9	24.2	0.089
Gestational age, wk	39.0 (1.4)	39.1 (1.4)	39.0 (1.6)	0.46
<38 wk gestation, %	18.4	17.9	21.9	0.68
Length, cm	48.7 (2.7)^a^	49.0 (2.7)^a^	48.1 (2.9)^b^	0.022
Head circumference, cm	34.3 (1.6)	34.2 (1.5)	33.8 (1.8)	0.079

Abbreviations: Nn, mother HIV negative, baby HIV negative; Pn, mother HIV positive, baby HIV negative at 6 months; Pp, mother HIV positive, baby HIV positive at 6 weeks.

^a,b ^Means in a row without a common letter differ, P < 0.05, Bonferroni multiple-comparison test.

* From analysis of variance for continuous variables and χ^2^-test for categorical variables.

† Values are mean (SD) or %.

‡ Variables (*n*) with missing data include maternal hemoglobin (21), CD4+ lymphocyte count (53), maternal age (1), parity (0), birthweight (3), gestational age (9), length (1), and head circumference (1).

Breastfeeding was nearly universal among infants in ZVITAMBO, but exclusive breastfeeding was rare [19]. Nonbreastmilk foods or liquids, were introduced to 90.2% of infants in the anemia substudy by 3 months and to 95.0% by 6 months, though very rarely did these foods include iron-rich sources, i.e., meat, poultry, eggs, fish, or iron-fortified infant formula. The common infant foods were juice and porridge made from maize meal and diluted with water. There were no statistically significant differences in patterns of infant feeding among the 3 HIV status groups.

### Anemia and iron status

**Figure 2:**
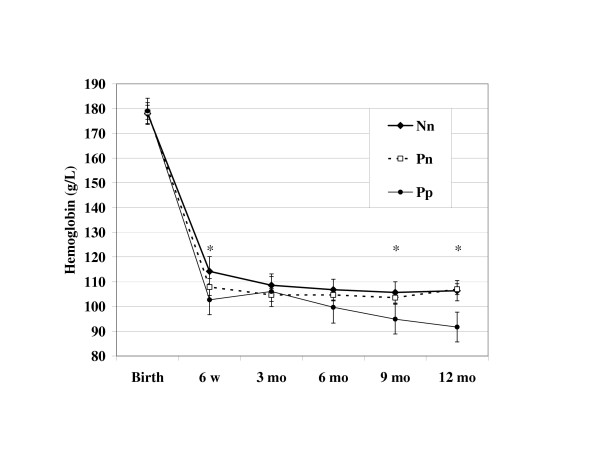
**Hemoglobin concentration during the first year of life in Zimbabwean babies by HIV status**. Values are means with 95% confidence intervals, adjusted for birthweight. The asterisk indicates a statistically significant difference in mean hemoglobin among the groups at a certain time point (6 weeks, F = 3.54, P = 0.030; 9 months, F = 4.37, P = 0.005; 12 months, F = 11.34, P < 0.001). Abbreviations: Nn, mother HIV negative, baby HIV negative; Pn, mother HIV positive, baby HIV negative at 6 months; Pp, mother HIV positive, baby HIV positive at 6 weeks.

The only difference in indicators of anemia and iron status between Nn and Pn babies was a significantly higher TfR concentration at 6 weeks of age in Pn babies (P = 0.001; P = 0.003, Bonferroni adjusted) (Table 2). In both HIV-infected and uninfected babies, mean hemoglobin concentration decreased dramatically from birth to 6 weeks, in a pattern consistent with the postnatal physiologic decline, but did not rise again (Figure 2). HIV-infected babies had the lowest 6 week concentration even after adjusting for birthweight (mean ± SE; 103 ± 3.0 g/L), significantly lower than that of babies born to HIV-negative mothers (114 ± 3.1 g/L, P = 0.008; P = 0.024, Bonferroni adjusted). From this nadir, hemoglobin slightly increased, but the improvement was not sustained beyond 3 months of age, after which hemoglobin progressively declined to significantly lower values than either Nn or Pn babies at 9 and 12 months of age. By 6 months, the odds of anemia (hemoglobin < 105 g/L) was more than two-fold greater among HIV-positive babies compared to HIV-negative babies (59.8% vs. 36.6%, adjusted for birthweight, P = 0.021). At 12 months, 36.6% of HIV-negative babies were anemic and 75.9% of HIV-positive babies (adjusted odds ratio = 5.45 (95% CI: 2.22, 13.4; P < 0.001) were anemic.

**Table 2:** Anemia and iron status in Zimbabwean infants from birth through 6 months of age by HIV status of the mother and her infant.

Indicator	Birth	6 weeks	3 months	6 months
HIV status group				
Serum ferritin, μg/L*				
Nn group	199 (172,230)^a,b^	131 (107,160)^a^	52 (44,62)^a^	15 (12,18)^a^
*n*	*108*	*63*	*64*	*75*
Pn group	198 (181,216)^a^	168 (153,185)^a^	62 (56,69)^a^	17 (15,19)^a^
*n*	*248*	*205*	*215*	*235*
Pp group	251 (211,300)^b^	320 (255,402)^b^	141 (104,191)^b^	36 (24,54)^b^
*n*	*76*	*70*	*52*	*40*
P-value^†^	0.036	<0.0001	<0.0001	<0.0001
Erythropoietin, U/L				
Nn group	----------	10.2 (8.5,12.2)^a^	12.2 (10.5,14.0)^a^	16.2 (13.7,19.0)^a^
*n*		*62*	*60*	*74*
Pn group		9.3 (8.4,10.2)^a^	11.6 (10.7,12.5)^a^	14.4 (13.0,15.8)^a^
*n*		*201*	*215*	*231*
Pp group		16.3 (13.4,19.9)^b^	23.4 (17.1,32.1)^b^	26.1 (20.0,34.0)^b^
*n*		*69*	*53*	*38*
P-value		<0.0001	<0.0001	<0.0001
Transferrin receptor, mg/L				
Nn group	----------	3.5 (3.2,3.8)^a^	6.2 (5.9,6.6)^a^	6.8 (6.3,7.3)
*n*		*63*	*64*	*75*
Pn group		4.1 (4.0,4.3)^b^	6.7 (6.4,7.0)^a^	7.2 (6.9,7.6)
*n*		*205*	*216*	*235*
Pp group		4.7 (4.3,5.2)^c^	8.2 (7.2,9.3)^b^	8.0 (7.0,9.1)
*n*		*71*	*53*	*40*
P-value		<0.0001	0.0001	0.065

Abbreviations: Nn, mother HIV negative, baby HIV negative; Pn, mother HIV positive, baby HIV negative at 6 months; Pp, mother HIV positive, baby HIV positive at 6 weeks.

^a,b,c ^Means in a cell without a common letter differ, P < 0.05, Bonferroni multiple-comparison test.

* Values are geometric mean (95% CI).

† P-values are from analysis of variance of log-transformed values.

Plasma ferritin concentration decreased from birth levels as expected with the physiologic mobilization of iron from liver stores in all HIV status groups. However, at all time points, including birth, the ferritin distribution was shifted to higher values among HIV-infected babies. For example, at 3 months, geometric mean [95% CI] ferritin concentration was significantly greater among HIV-positive infants (140.7 [103.8, 190.7], n = 52) compared to HIV-negative infants (59.8 [54.5,65.6], n = 279; P < 0.0001). At 6 months, serum ferritin was not associated with hemoglobin in any HIV status group. Geometric mean plasma EPO increased with age and TfR increased to 3 months then leveled off (see Table 2). Compared to HIV-negative babies, mean EPO concentration was significantly higher among HIV-positive infants at 6 weeks, 3 months, and 6 months, and mean TfR was higher at 6 weeks and 3 months.

### Relation of EPO to anemia

Among Nn and Pn infants, the slope of the fitted regression line between log_10 _plasma EPO (U/L) and hemoglobin (i.e., the EPO response to anemia) was significantly negative at 6 weeks; this association remained at 3 months and 6 months but was only significant among the Pn infants (see Additional file 1). Among Pp infants, the EPO response to anemia was attenuated at 6 weeks and 3 months, but significant at 6 months. Among Nn babies, the EPO response to anemia became weaker with age, while among Pn and Pp babies, the response was stronger at 6 months than at 6 weeks or 3 months.

### Relation of TfR to EPO

TfR and EPO were positively associated at all ages and for all HIV status groups. A significant interaction with HIV status was observed at 3 months of age. The increase in log_e _plasma TfR (mg/L) for one log_10 _decrease in plasma EPO (U/L) was significantly greater for Pp babies compared to Pn babies (P = 0.023), and there was a trend for a greater increase when compared to Nn babies (P = 0.11).

### Relation of ferritin to TfR

There was no consistent or significant relation between ferritin and TfR at 6 weeks or 3 months. However, there was a significant interaction between HIV status and TfR on ferritin concentration at 6 months (P = 0.01). Ferritin was inversely associated with TfR in HIV-negative infants born to HIV-positive and HIV-negative mothers but not in HIV-positive infants.

### Variability in TfR

To compare the relative strength of association between TfR and ferritin vs. EPO, we examined the partial correlation coefficients between these variables. TfR was more strongly correlated with EPO than ferritin in all HIV status groups at every age, after adjusting for hemoglobin. There was a strong inverse association between TfR and ferritin only at 6 months in Pn infants, but, even then, more of the variability in TfR was explained by EPO concentration than ferritin.

## Discussion

In this study of Zimbabwean babies, anemia was prevalent and strongly influenced by HIV infection. Though HIV infected and uninfected infants had similar hemoglobin distributions at birth, by 12 months of age infected infants had a nearly 20 g/L lower mean hemoglobin and they were >5 times as likely to be anemic, even after adjusting for their lower birthweight.

Our model of the relationships between indicators of iron status and erythropoiesis proved valid among HIV-negative infants. The data support the hypothesized relationships between indicators of erythropoiesis: EPO was inversely associated with hemoglobin and positively associated with TfR; TfR was inversely associated with ferritin (although weakly at 6 weeks and 3 months); and EPO explained more variability than ferritin in TfR concentration.

HIV infection perturbed the normal feedback system and modified some, but not all, of the hypothesized relationships. The EPO response to anemia among HIV-infected infants was attenuated during early infancy but normalized at 6 months of age. HIV infection did not seem to affect the bone marrow response to stimulation by EPO, i.e., TfR increased in direct response to increased EPO levels, at all ages. However at 6 months, this drive in red blood cell production was not accompanied by the expected decline in ferritin in HIV-infected infants.

A limitation of the present study was the high rate of missing values for indicators of anemia and erythropoiesis. Infant blood samples were unavailable if the mother-baby pair defaulted, the infant died, or if the blood sample was insufficient. It is possible that these losses could have biased the results, but there were no statistically significant differences in any measured baseline maternal factor between infants followed versus not followed. We did observe a statistically significant difference in preterm birth between infants who came for the 6 month visit and those who did not, but the impact of preterm on the likelihood of missing values was similar across HIV status groups and likely did not bias the results. Insufficient blood sample as a result of either inadequate quantity or quality, e.g., the sample clotted before hemoglobin was measured) was not associated with HIV status. Finally, survival rates were certainly associated with infant HIV status with approximately half of HIV-positive infants dead by 6 months, but very few deaths among uninfected infants. Thus a survival bias is possible and should be considered in light of the study findings.

### Iron deficiency

One-third of HIV-negative infants had serum ferritin concentration <12 μg/L at 6 months suggesting that their anemia was largely caused by iron deficiency. Worldwide, the primary cause of anemia among infants is iron deficiency [29]. Infants have a relatively high demand for iron because they are rapidly growing, and human milk is low in iron, even after accounting for good bioavailability. In most developing countries, complementary food diets are largely plant-based and high in substances that inhibit iron absorption and are by themselves insufficient to meet dietary needs for iron [30-32]. Foods with naturally high iron content and high bioavailability, such as meat, and iron-fortified infant formula are rarely consumed. We too observed these infant-feeding patterns in the present study. Furthermore, ~one-third of the mothers of HIV-uninfected infants were anemic (hemoglobin <110 g/L) at delivery, which is also a risk factor for infant anemia [33,34].

In the absence of concurrent disease, serum ferritin parallels total body iron stores and can reflect deficient, normal, and excess iron stores [27]. However, ferritin is also a positive acute phase protein and can rise in the presence of infection or inflammation [35]. Among the HIV-positive infants in this study, we conclude that serum ferritin more strongly reflected concurrent infection than iron status because: a) it was consistently higher among HIV-infected infants compared to HIV-negative infants; and, b) it was inversely associated with TfR among uninfected infants but not among HIV positive infants. A measure of immune activation would have provided further clarification and should be incorporated into future studies.

### Inadequate EPO response

Our findings that the EPO response to anemia was blunted in HIV-infected babies at 6 weeks and slightly attenuated at 3 months of age are consistent with several reports of studies carried out in HIV-infected adults [6,9,36]. This attenuation is thought to be through inhibition by inflammatory cytokines, such as interleukin-1 and tumor necrosis factor, or EPO gene transcription [10,37,38]. However, by 6 months of age, the EPO response to anemia was not different than that observed among uninfected infants. In fact, mean log_10 _EPO concentration was highest in Pp infants at all ages, even after adjusting for hemoglobin concentration (data not shown). To our knowledge, the only other study, in addition to the present, to compare EPO in HIV-infected and uninfected infants was carried out in Malawi, and they too did not show an inadequate EPO response among HIV-infected infants [11]. It is noteworthy that the infants in the Malawi study were 12 months-old. They, like the babies in the present study at 6 months of age, represent a different stage in physiological development than 6 week old infants. It is not surprising that the age of the infant plays a crucial role in the regulation of erythropoiesis and perturbation to the system. These ages span highly significant changes in normal hematological development characterized by a switch in the site of EPO production from the liver to the kidney, greater oxygen consumption with increasing activity and growth, and a switch from fetal to adult hemoglobin with a decrease in oxygen affinity favoring oxygen delivery.

The apparent transient impact of HIV on the EPO response to anemia might suggest that the attenuating effect on EPO is more pronounced closer to the time of initial infection and weakens with progression of HIV disease. Acute HIV infection is characterized by a peak in viremia and rapid decline in CD4+ cells, but an immunologic response ensues resulting in a lowering of the plasma level of HIV RNA to a steady state "set point" that reflects a balance between production and destruction of virions [39]. It is possible that we have observed this steady state in HIV infected infants at 6 months of age. Alternatively, the higher mean EPO levels in the Pp group may be explained by dyserythropoiesis (due to a direct effect of HIV and/or through cytokine activation) or reduced number of stem cells in HIV infected infants. However, we observed a strong relationship between EPO and TfR suggesting that bone marrow cells are responding to stimulation by EPO. A measure of reticulocyte count would be helpful to clarify this mechanism and should be considered in future studies of infant hematopoiesis. Finally, we speculate that the stronger EPO response observed in Pp infants at 6 months may in part be attributed to a survival bias. We compared the EPO-Hb slope among Pp infants who died by 12 months of age to that of Pp infants who survived to 12 months. Only 2 infants who died in the first year had both EPO and Hb measurements at 6 months of age making it impossible to estimate the slope in this group at 6 months. A comparison of the slopes at 3 months of age showed a trend for a flatter slope among those who died (+0.0072) than among those who survived (-0.0017) suggesting greater attenuation of the EPO-Hb association among those who died and, perhaps, unique resilience among those who survived. On the other hand, the slope at 6 weeks was steeper among infants who died (-0.0042) than among those who survived (-0.0025) and more like that observed among Pn infants (-0.0044). This post hoc analysis was limited by sample size, and differences among HIV-infected infants who survive and among those who die should be further explored.

It is important to note that the inverse association between EPO and hemoglobin was not statistically significant in Nn babies at 3 and 6 months of age. The failure to reach statistical significance may in part be attributed to a lack of sample size since slopes of similar magnitude in the Pn group were statistically significant. However, the data also suggest a relatively weak inverse association between these indicators. In fact, the observed slopes of the regression line between log_10 _EPO and hemoglobin (g/L), even the steepest observed value (-0.0109) in Pp infants at 6 months, were less steep than values previously reported in the literature. The values of slopes for control groups comprised of HIV-uninfected infants [11], children with iron deficiency anemia [40] and sickle cell disease [41], and children with hematological disease [42] have ranged from -0.011 to -0.0553. Young children in developing countries are often sick, with the prevalence of diarrhea ranging from 10 to 19 percent [43-45], and the prevalence of acute respiratory illness among young children in developing countries even higher, 25 to 60 percent [46]. We speculate that the inflammatory response to common childhood infections may have caused an attenuation of the EPO response to anemia. Additionally, vitamin A has been shown to stimulate EPO production in cell culture and animal models [47-50]. Thus, it is possible that vitamin A deficiency might have reduced the EPO response to low hemoglobin concentrations.

### Impaired iron mobilization

A noteworthy effect of HIV infection on erythropoiesis was the modification of infant infection on the association between ferritin and TfR at 6 months of age. The absence of a negative association between TfR and serum ferritin in HIV-infected babies may be explained by confounding due to infection, yet it is consistent with the sequestration of iron in liver stores during infection. The concurrent lack of association at 6 months between ferritin and hemoglobin supports this explanation since an increase in ferritin did not translate into increased hemoglobin production. However, these results should be interpreted with caution since serum ferritin is an acute phase protein and elevated during infection and inflammation [35] at which times the indicator has limited ability to accurately reflect iron stores. The lack of association between ferritin and hemoglobin that we observed at 6 months in all HIV status groups supports the notion that ferritin levels do not specifically reflect iron stores but are also affected by infection status.

### TfR as an indicator of erythropoiesis

The observation that at all ages and in all HIV groups, EPO concentration explained more variability in TfR levels than did serum ferritin concentration suggests that TfR is not primarily an indicator of iron deficiency in this age group. We speculate that during periods of high erythropoietic activity (e.g. early infancy), an increase in TfR more strongly reflects an increase in erythroblasts (i.e. erythropoiesis) rather than an increase in the density of receptors per red blood cell consistent with tissue iron deficiency. While the strong and consistent association between TfR and EPO is striking, particularly when compared to the lack of association between TfR and ferritin, it was not surprising that ferritin did not explain much of the variability in TfR given its association with infection and inflammation.

## Conclusion

We conclude that HIV-related anemia is associated with attenuation in the EPO response to anemia in early infancy, but that the EPO response is normalized by 6 months. Throughout the first 6 months of infancy, HIV-infected babies have higher serum ferritin concentrations, which could reflect iron sequestration in liver stores, or an acute phase response. If the elevated serum ferritins in infected babies are explained by inflammation, then the HIV-related anemia might be explained by a shortened red cell lifespan [12] or by increased hepatic hepcidin production leading to downregulated intestinal iron absorption and retention of iron in reticuloendothelial macrophages, causing iron deficient erythropoiesis [51]. These hypotheses should be investigated in future research. With regard to assessment of iron status, we found that the serum ferritin distribution was shifted significantly to the right in HIV-infected babies even at birth, and that TfR behaved primarily as an indicator of erythropoiesis in all infants. Both indicators should be used cautiously as indicators of iron deficiency in babies.

## List of abbreviations used

Nn, mother HIV negative, baby HIV negative; Pn, mother HIV positive, baby HIV negative at 6 months; Pp, mother HIV positive, baby HIV positive at 6 weeks; EPO, erythropoietin; TfR, transferrin receptor; HIV, human immunodeficiency virus; ZVITAMBO, The Zimbabwe Vitamin A for Mothers and Babies Project.

## Competing interests

The author(s) declare that they have no competing interests.

## Authors' contributions

MFM contributed to the design of the study, carried out the research, performed the statistical analyses and wrote the manuscript. RJS and JHH contributed to the design of the study, conduct of the research, interpretation of the results, and assisted in writing the manuscript. PJI, LCM, and NVM assisted in the conduct of the research and editing of the manuscript.

## Pre-publication history

The pre-publication history for this paper can be accessed here:



## Supplementary Material

Additional File 1 Table 3. Slope of the regression line for indicators of erythropoiesis (i.e., β-coefficients) by age and maternal and infant HIV status Click here for file

## References

[B1] UNAIDS (2004). 2004 Report on the global AIDS epidemic. http://www.unaids.org/bangkok2004/report.html.

[B2] Gray L, Newell ML, Thorne C, Peckham C, Levy J (2001). Fluctuations in symptoms in human immunodeficiency virus-infected children: the first 10 years of life. Pediatrics.

[B3] Sullivan PS, Hanson DL, Chu SY, Jones JL, Ward JW (1998). Epidemiology of anemia in human immunodeficiency virus (HIV)-infected persons: results from the multistate adult and adolescent spectrum of HIV disease surveillance project. Blood.

[B4] Moore RD, Keruly JC, Chaisson RE (1998). Anemia and survival in HIV infection. J Acquir Immune Defic Syndr Hum Retrovirol.

[B5] Kreuzer KA, Rockstroh JK (1997). Pathogenesis and pathophysiology of anemia in HIV infection. Ann Hematol.

[B6] Spivak JL, Barnes DC, Fuchs E, Quinn TC (1989). Serum immunoreactive erythropoietin in HIV-infected patients. JAMA.

[B7] Semba RD, Gray GE (2001). Pathogenesis of anemia during human immunodeficiency virus infection. J Investig Med.

[B8] Jelkmann W, Pagel H, Wolff M, Fandrey J (1992). Monokines inhibiting erythropoietin production in human hepatoma cultures and in isolated perfused rat kidneys. Life Sci.

[B9] Kreuzer KA, Rockstroh JK, Jelkmann W, Theisen A, Spengler U, Sauerbruch T (1997). Inadequate erythropoietin response to anaemia in HIV patients: relationship to serum levels of tumour necrosis factor-alpha, interleukin-6 and their soluble receptors. Br J Haematol.

[B10] Wang Z, Goldberg MA, Scadden DT (1993). HIV-1 suppresses erythropoietin production in vitro. Exp Hematol.

[B11] Semba RD, Broadhead R, Taha TE, Totin D, Ricks MO, Kumwenda N (2001). Erythropoietin response to anemia among human immunodeficiency virus-infected infants in Malawi. Haematologica.

[B12] Spivak JL (2000). The blood in systemic disorders. Lancet.

[B13] Spivak JL, Pham T, Isaacs M, Hankins WD (1991). Erythropoietin is both a mitogen and a survival factor. Blood.

[B14] Koury MJ, Bondurant MC (1990). Erythropoietin retards DNA breakdown and prevents programmed death in erythroid progenitor cells. Science.

[B15] Kohgo Y, Niitsu Y, Kondo H, Kato J, Tsushima N, Sasaki K, Hirayama M, Numata T, Nishisato T, Urushizaki I (1987). Serum transferrin receptor as a new index of erythropoiesis. Blood.

[B16] Beguin Y, Clemons GK, Pootrakul P, Fillet G (1993). Quantitative assessment of erythropoiesis and functional classification of anemia based on measurements of serum transferrin receptor and erythropoietin. Blood.

[B17] Skikne BS, Flowers CH, Cook JD (1990). Serum transferrin receptor: a quantitative measure of tissue iron deficiency. Blood.

[B18] Malaba LC, Iliff PJ, Nathoo KJ, Marinda E, Moulton LH, Zijenah LS, Zvandasara P, Ward BJ, Humphrey JH (2005). Effect of postpartum maternal or neonatal vitamin A supplementation on infant mortality among infants born to HIV-negative mothers in Zimbabwe. Am J Clin Nutr.

[B19] Iliff PJ, Piwoz EG, Tavengwa NV, Zunguza CD, Marinda ET, Nathoo KJ, Moulton LH, Ward BJ, Humphrey JH (2005). Early exclusive breastfeeding reduces the risk of postnatal HIV-1 transmission and increases HIV-free survival. AIDS.

[B20] Humphrey JH, Iliff PJ, Marinda E, Mutasa K, Moulton LH, Nathoo KJ, Chidawanyika H, Ward BJ, Malaba LC, Zijenah LS, Zvandasara P, Mahomva A, Ruff A, Mbizvo MT, Zunguza C (2005). Impact of single large doses of vitamin A given during the postpartum period to HIV-infected women and their neonates on breastfeeding-associated mother-to-child-transmission of HIV and infant mortality in Zimbabwe. JID.

[B21] Capurro H, Konichezky S, Fonseca D, Caldeyro-Barcia R (1978). A simplified method for diagnosis of gestational age in the newborn infant. J Pediatr.

[B22] WHO/UNICEF/UNU (1997). Iron deficiency: indicators for assessment and strategies for prevention.

[B23] Michaelsen KF, Milman N, Samuelson G (1995). A longitudinal study of iron status in healthy Danish infants: effects of early iron status, growth velocity and dietary factors. Acta Paediatr.

[B24] Emond AM, Hawkins N, Pennock C, Golding J (1996). Haemoglobin and ferritin concentrations in infants at 8 months of age. Arch Dis Child.

[B25] Sherriff A, Emond A, Hawkins N, Golding J (1999). Haemoglobin and ferritin concentrations in children aged 12 and 18 months. ALSPAC Children in Focus Study Team. Arch Dis Child.

[B26] Domellof M, Dewey KG, Lonnerdal B, Cohen RJ, Hernell O (2002). The diagnostic criteria for iron deficiency in infants should be reevaluated. J Nutr.

[B27] Dallman PR, Nathan DG and Oski FA (1993). Iron deficiency and related nutritional anemias. Hematology of Infancy and Childhood.

[B28] Lipschitz DA, Cook JD, Finch CA (1974). A clinical evaluation of serum ferritin as an index of iron stores. N Engl J Med.

[B29] Stoltzfus RJ (2003). Iron deficiency:  global prevalence and consequences. Food Nutr Bull.

[B30] WHO/UNICEF (1998). Complementary feeding of young children in developing countries: a review of current scientific knowledge.

[B31] Gibson RS, Ferguson EL, Lehrfeld J (1998). Complementary foods for infant feeding in developing countries: their nutrient adequacy and improvement. Eur J Clin Nutr.

[B32] Dewey KG, Brown KH (2003). Update on technical issues concerning complementary feeding of young children in developing countries and implications for intervention programs. Food Nutr Bull.

[B33] De Pee S, Bloem MW, Sari M, Kiess L, Yip R, Kosen S (2002). The high prevalence of low hemoglobin concentration among Indonesian infants aged 3-5 months is related to maternal anemia. J Nutr.

[B34] Miller MF, Stoltzfus RJ, Mbuya NV, Malaba LC, Iliff PJ, Humphrey JH (2003). Total body iron in HIV-positive and HIV-negative Zimbabwean newborns strongly predicts anemia throughout infancy and is predicted by maternal hemoglobin concentration. J Nutr.

[B35] Worwood M (1997). The laboratory assessment of iron status--an update. Clin Chim Acta.

[B36] Camacho J, Poveda F, Zamorano AF, Valencia ME, Vazquez JJ, Arnalich F (1992). Serum erythropoietin levels in anaemic patients with advanced human immunodeficiency virus infection. Br J Haematol.

[B37] Jelkmann W, Wolff M, Fandrey J (1990). Modulation of the production of erythropoietin by cytokines: in vitro studies and their clinical implications. Contrib Nephrol.

[B38] Faquin WC, Schneider TJ, Goldberg MA (1992). Effect of inflammatory cytokines on hypoxia-induced erythropoietin production. Blood.

[B39] Burger S, Poles MA (2003). Natural history and pathogenesis of human immunodeficiency virus infection. Semin Liver Dis.

[B40] Bray GL, Taylor B, O'Donnell R (1992). Comparison of the erythropoietin response in children with aplastic anemia, transient erythroblastopenia, and iron deficiency. J Pediatr.

[B41] Corazza F, Beguin Y, Bergmann P, Andre M, Ferster A, Devalck C, Fondu P, Buyse M, Sariban E (1998). Anemia in children with cancer is associated with decreased erythropoietic activity and not with inadequate erythropoietin production. Blood.

[B42] Eckardt KU, Hartmann W, Vetter U, Pohlandt F, Burghardt R, Kurtz A (1990). Serum immunoreactive erythropoietin of children in health and disease. Eur J Pediatr.

[B43] Tomkins A (1981). Nutritional status and severity of diarrhoea among pre-school children in rural Nigeria. Lancet.

[B44] Molbak K, Wested N, Hojlyng N, Scheutz F, Gottschau A, Aaby P, da Silva AP (1994). The etiology of early childhood diarrhea: a community study from Guinea-Bissau. J Infect Dis.

[B45] Rowland MG, Rowland SG, Cole TJ (1988). Impact of infection on the growth of children from 0 to 2 years in an urban West African community. Am J Clin Nutr.

[B46] Black RE, Brown KH, Becker S, Alim AR, Huq I (1982). Longitudinal studies of infectious diseases and physical growth of children in rural Bangladesh. II. Incidence of diarrhea and association with known pathogens. Am J Epidemiol.

[B47] Jelkmann W, Pagel H, Hellwig T, Fandrey J (1997). Effects of antioxidant vitamins on renal and hepatic erythropoietin production. Kidney Int.

[B48] Okano M, Masuda S, Narita H, Masushige S, Kato S, Imagawa S, Sasaki R (1994). Retinoic acid up-regulates erythropoietin production in hepatoma cells and in vitamin A-depleted rats. FEBS Lett.

[B49] Neumcke I, Schneider B, Fandrey J, Pagel H (1999). Effects of pro- and antioxidative compounds on renal production of erythropoietin. Endocrinology.

[B50] Kambe T, Tada-Kambe J, Kuge Y, Yamaguchi-Iwai Y, Nagao M, Sasaki R (2000). Retinoic acid stimulates erythropoietin gene transcription in embryonal carcinoma cells through the direct repeat of a steroid/thyroid hormone receptor response element half-site in the hypoxia-response enhancer. Blood.

[B51] Vyoral D, Petrak J (2005). Hepcidin: a direct link between iron metabolism and immunity. Int J Biochem Cell Biol.

