# Supplementary material for: Neonatal erythropoiesis and subsequent anemia in HIV-positive and HIV-negative Zimbabwean babies during the first year of life: a longitudinal study
Source: BMC Infect Dis. 2006 Jan 3;6:1. doi: 10.1186/1471-2334-6-1 (PMC1361802; doi:10.1186/1471-2334-6-1)
Supplement: Additional File 1 — Table 3. Slope of the regression line for indicators of erythropoiesis (i.e., β-coefficients) by age and maternal and infant HIV status [file 1471-2334-6-1-S1.doc]

**Table 3**. Slope of the regression line for indicators of erythropoiesis (i.e., β-coefficients) by age and maternal and infant HIV status.

|  | HIV status group | | | Comparison of β-coefficients* | | |
| --- | --- | --- | --- | --- | --- | --- |
|  | Nn | Pn | Pp | Nn vs. Pn | Nn vs. Pp | Pn vs. Pp |
| Log_10_ EPO and hemoglobin |  |  |  |  |  |  |
| 6 weeks^†^ | β= −0.0086 (−0.0134, −0.0038)  R=−0.493, P=0.001  N=43 | β= −0.0044 (−0.0067, −0.0022)  R=−0.314, P<0.001  N=144 | β= −0.0024 (−0.0061, 0.0013)  R=−0.191, P=0.204  N=46 | P=0.15 | P=0.049 | P=0.31 |
| 3 months | β= −0.0051 (−0.0114, 0.0012)  R=−0.251, P=0.109  N=42 | β= −0.0030 (−0.0051, −0.0008)  R=−0.220, P=0.007  N=147 | β= 0.0021 (−0.0141, 0.0182)  R=0.054, P=0.793  N=26 | P=0.61 | P=0.23 | P=0.28 |
| 6 months | β= −0.0037 (−0.0093, 0.0018)  R=−0.181, P=0.181  N=56 | β= −0.0075 (−0.0101, −0.0049)  R=−0.393, P<0.001  N=176 | β= −0.0109 (−0.0197, −0.0021)  R=−0.473, P=0.017  N=25 | P=0.24 | P=0.13 | P=0.39 |
| Log_e_ TfR and log_10_ EPO |  |  |  |  |  |  |
| 6 weeks | β= 0.5613 (0.3124, 0.8103)  R=0.503, P<0.001  N=62 | β= 0.4602 (0.3226, 0.5977)  R=0.425, P<0.001  N=199 | β= 0.6269 (0.4076, 0.8462)  R=0.572, P<0.001  N=69 | P=0.48 | P=0.68 | P=0.18 |
| 3 months | β= 0.3231 (0.0781, 0.5682)  R=0.327, P=0.011  N=60 | β= 0.3509 (0.1726, 0.5292)  R=0.260, P<0.001  N=215 | β= 0.6329 (0.4430, 0.8228)  R=0.684, P<0.001  N=53 | P=0.89 | P=0.11 | P=0.023 |
| 6 months | β= 0.4644 (0.2397, 0.6890)  R=0.437, P<0.001  N=74 | β= 0.5478 (0.4296, 0.6661)  R=0.517, P<0.001  N=231 | β= 0.4864 (0.1198, 0.8530)  R=0.409, P=0.011  N=38 | P=0.53 | P=0.91 | P=0.69 |
| Log_e_ ferritin and log_e_ TfR |  |  |  |  |  |  |
| 6 weeks | β= −0.4251 (−1.0036, 0.1534)  R=–0.185, P=0.147  N=63 | β= −0.2296 (−0.5271, 0.0678)  R=–0.106, P=0.130  N=204 | β= −0.2560 (−0.8457, 0.3336)  R=–0.104, P=0.389  N=70 | P=0.55 | P=0.65 | P=0.93 |
| 3 months | β= 0.0340 (−0.6734, 0.7414)  R=0.010, P=0.924  N=64 | β= −0.2667 (−0.5889, 0.0556)  R=–0.111, P=0.104  N=215 | β= 0.2624 (−0.4032, 0.9280)  R=0.111, P=0.432  N=52 | P=0.52 | P=0.65 | P=0.084 |
| 6 months | β= −0.8412 (−1.5416, −0.1408)  R=–0.270, P=0.019  N=75 | β= −0.7767 (−1.0957, −0.4576)  R=–0.300, P<0.001  N=235 | β= 0.3984 (−0.5760, 1.3729)  R=0.133, P=0.413  N=40 | P=0.86 | P=0.012 | P=0.003 |
| Log_e_ ferritin and hemoglobin |  |  |  |  |  |  |
| 6 months | β= 0.0120 (−0.0074, 0.0314)  R=0.165, P=0.219  N=75 | β= 0.0038 (−0.0041, 0.0117)  R=0.071, P=0.349  N=235 | β= 0.0081 (−0.0272, 0.0434)  R=0.094, P=0.640  N=27 | P=0.43 | P=0.80 | P=0.74 |

Abbreviations: Nn, mother HIV negative, baby HIV negative; Pn, mother HIV positive, baby HIV negative at 6 months; Pp, mother HIV positive, baby HIV positive at 6 weeks; EPO, erythropoietin; TfR, transferrin receptor.

* P-value for interaction between the HIV status group and the indicator of erythropoiesis (the independent variable) in regression analysis.

† Values are β-coefficient (95% confidence interval). R is Pearson’s correlation coefficient, and P is the P-value from the bivariate linear regression model.
